# CYTOP® 366: A Tertiary Phosphine Inaccessible by Most Traditional Hydrophosphination Methods

**DOI:** 10.1002/open.202300264

**Published:** 2024-01-03

**Authors:** Dino Amoroso, Jeff Dyck, Andrew Jackson, Michael Humeniuk, Eleanor Kendrick, Angelo Melaragni, Michael Moser, Izabela Wiater‐Protas, Serguei Zavorine, Jade Markham

**Affiliations:** ^1^ Phosphorus Specialties, Technology Solutions Syensqo 9061 Garner Road Niagara Falls ON, L2H 0Y2 Canada; ^2^ Research and Innovation Technology Solutions Syensqo 1937 West Main Street Stamford CT 06902 USA; ^3^ Phosphorus Specialties, Technology Solutions Syensqo Trinity Street B69 4LN Oldbury UK

**Keywords:** Tricyclohexylphosphine, Hydrophosphination, CYTOP® 366, Michael Addition, Tertiary Phosphine

## Abstract

Homogenous catalysis is an essential tool within the commercial manufacture of bulk and fine chemicals. Within this, phosphine ligands, such as tricyclohexylphosphine, otherwise known as CYTOP® 366, are a crucial component. When designing a pathway to your ligand of choice, some key considerations include safety, yield and quality, but at commercial volumes we must also balance cost and consider the technologies readily available. Herein, we report the synthetic route that was chosen to manufacture tricyclohexylphosphine at commercial scale. We also consider, with the use of computational calculations, why traditional hydrophosphination methods failed, where the selected pathway succeeded.

## Graphical abstract

Traditional radical‐catalyzed methods to prepare alkylphosphines are not suitable for the target molecule, tricyclohexylphosphine, otherwise known as CYTOP® 366. Through computational study, we explore why this tertiary phosphine is inaccessible via a radical‐catalyzed addition and describe an alternative pathway to our target, which not only achieves good yield and purity but is also a safe and workable route for industrial scale manufacture.

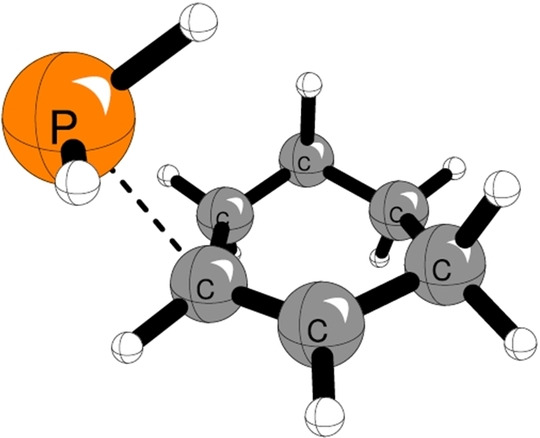

## Introduction

Phosphine ligands and their subsequent metal complexes are synonymous with homogenous catalysis. The tunability of a catalyst through the modification of the steric and electronic properties of its respective phosphine ligand, makes the use of phosphine ligands highly desirable, with small structural changes impacting yield and selectivity, in some cases, to large extents. Phosphine ligands are utilized throughout the chemical industry. Examples include Wilkinson's catalysts which are involved in reactions such as the hydrogenation of olefins.^[1]^

Within industry, although there is always a drive to develop the next generation ligand and expand the ligand portfolio, it is also important to revisit manufacturing processes and evaluate if it is possible to make a product in a better way. This may be driven by sustainability – finding routes that use renewable reagents, using a less energy intensive process, or employing starting materials more locally sourced – or through the increased awareness of health and safety implications, or cost. Additionally, internal factors such as access to specific equipment or assets can also drive the development of new processes.

**Figure 1:**
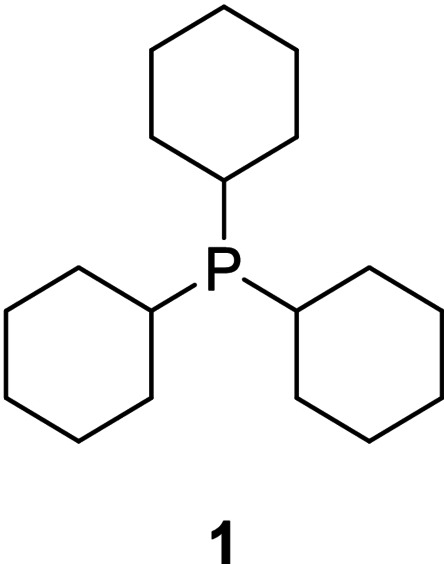
Chemical structure of tricyclohexylphosphine (**1**).

Tricyclohexylphosphine (**1**), known commercially as CYTOP® 366, is an important ligand used in a plethora of metal‐catalyzed organic transformations. This includes its use as a constituent of Grubbs catalysts which are employed in metathesis reactions and its use within Crabtree's catalysts used for hydrogenation reactions.[^2^, ^3^] The simple chemical structure of compound **1**, shown in Figure 1, may lead a chemist to assume its synthesis at industrial scale is equally straightforward, however manufacture at scale is not without its difficulties. In this paper, we will share an alternative route to compound **1** and the challenges that were overcome to achieve a safe, low‐cost and high‐yielding route, workable at commercial scale.

## Results and Discussion

A well‐known route to compound **1** was reported in 1954 by Issleib and Brack using phosphorus trichloride and cyclohexylmagnesium bromide.^[4]^ Although this synthetic pathway is used industrially and many chemical manufacturers routinely use Grignard chemistry, there are downsides that encouraged our investigations into alternative routes. These disadvantages include the need for low temperatures to control the highly exothermic reaction, which in turn also means the process is energy intensive. Rigorous safety requirements are necessary, as there are multiple avenues for error which can lead to a catastrophic event. An incident in Jiangsu province in 2014 resulted from the formation of gaseous alkanes after the reagents underwent rapid hydrolysis following a condenser leak. This led to both injury to a number of parties and a fatality.^[5]^

Other synthetic routes to compound **1** have been described in the literature. These include the hydrogenation of triphenylphosphine (TPP)^[6]^ and the direct synthesis of compound **1** from white and red phosphorus.^[7]^ Described literature pathways offer inspiration for manufacturing routes, but the process must correlate to the capabilities available and what is practical to do at scale.

### Free Radical Addition and Our Alternative

**Scheme 1:**
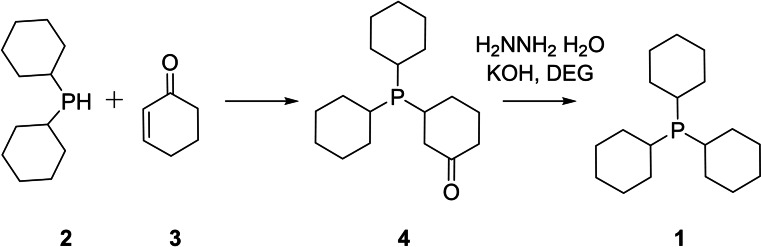
Synthesis of tricyclohexylphosphine **1** from secondary phosphine **2** and unsaturated enone **3**.

Radical‐catalyzed addition of olefins to phosphine is a long‐known route to forming P−C bonds.^[1]^ The route is often high‐yielding with few by‐products. Unfortunately, for sterically‐encumbered targets such as (**1**), it is often the case that formation of trialkylphosphines is not feasible under radical conditions.^[8]^ This is indeed the case as only the secondary phosphine, dicyclohexylphosphine (**2**), otherwise known as CYTOP® 266, is observed under typical radical‐catalyzed conditions. As a result, the route described herein (Scheme 1), leveraging the addition of the secondary phosphine (**2**) to the unsaturated enone (**3**) was explored.

The addition of secondary phosphines to unsaturated enones has been known for many years.^[9]^ Thus the addition of dicyclohexylphosphine (**2**) to cyclohexenone was evaluated as a potential means to install what would ultimately become the third cyclohexyl group of the target molecule (**1**). As expected, 2‐cyclohexen‐1‐one reacts with dicyclohexylphosphine, in the absence of any solvent, to provide the ketone‐substituted trialkylphosphine **4**. While a mild exotherm was noted upon addition of the enone to **2** (ΔH_rxn_=−13.56 kcal/mol; calculated at 298 K and 1 atm), heating to 95 °C for 16 hours followed by removal of volatiles leads to the isolation of the **4** in 87 % yield. At elevated temperatures the reaction product is a clear yellow liquid, and upon cooling to ambient it is a straw‐colored solid.

Other than removal of volatiles, the intermediate (**4**) was not purified further so as to simplify the overall process for **1**. Thus, the material remaining after removal of volatiles was subjected to the next stage of the process. Reaction of **4** with hydrazine proceeds quickly which is followed by reduction with potassium hydroxide over the course of several hours followed by removal of water via the Dean Stark apparatus. After dissolution in toluene and extraction with water, the organic phase was reduced to dryness leaving behind a pale yellow solid. Analysis of the residue by ^31^P NMR spectroscopy showed the product **1** had formed in approximately 96 % purity with the balance being the oxide.

The relative high purity observed by ^31^P NMR spectroscopy was considered unreliable owing to the yellow coloration of the solid and subsequent orange solution formed when the solid was dissolved. As such, other methods were explored to gauge the purity of the product. GC analysis was not helpful in identifying any impurities that may be present leading to the strong coloration as few volatile impurities were detected. As such, a thermogravimetric analysis of the solid was conducted. The material derived was found to contain a significant amount of high boiling impurities, >250–300 °C, as almost 20 % of the derived solid persisted beyond this range. Comparison to a known good sample showed complete volatilization of the solid just below 240 °C.

In order to improve the purity of the product derived from this process, a recrystallization was conducted. Thus, a saturated toluene solution of **1** was recrystallized from acetonitrile/methanol at 5 °C. The resulting white precipitate was filtered and dried under vacuum to give a white solid in 97 % yield. The solid was found to be >98 % purity by ^31^P NMR and was free of any high‐boiling impurities by TGA.

### Computational Chemistry

Simulations were performed to investigate potentially discernable mechanistic differences between the radical‐mediated pathway and the two‐electron Michael addition pathway.^[10]^

Schrödinger's Jaguar quantum chemistry module version 23‐2 was used for performing DFT calculations on the radical alkylation steps for addition of cyclohexene to each radical P species. For comparison we also show the corresponding additions for synthesizing triethylphosphine (PEt_3_) also via radical chemistry because this reaction system proceeds with high yields. In addition, we show the third alkylation step using the Michael addition pathway in which neutral dicyclohexylphosphine (HPCy_2_) is reacted with cyclohexenone in diethylene glycol. Calculations to depict radical formation, transfer, or termination were not performed.

Geometry optimization on reactants and intermediates was conducted using the M06‐2X (AJ1) functional with def2‐TZVP (AJ2) as basis set. Vibrational frequencies and thermochemical data were obtained using single point calculations with def2‐TZVP (‐F) basis set due to limitations of f‐orbitals in calculating frequencies.

Transition states were identified with the functional and basis set listed above for the geometry optimization and subsequently verified with a single point energy calculation showing a lone negative frequency along the reaction coordinate depicting the formation of the phosphorus carbon bond.

An implicit solvation model of toluene (AJ3) was used for all calculations involving the radical alkylation pathways (i. e. cyclohexene and ethylene). Methanol was used as an implicit solvent for the Michael addition step, which has a dielectric constant similar to diethylene glycol.

**Figure 2:**
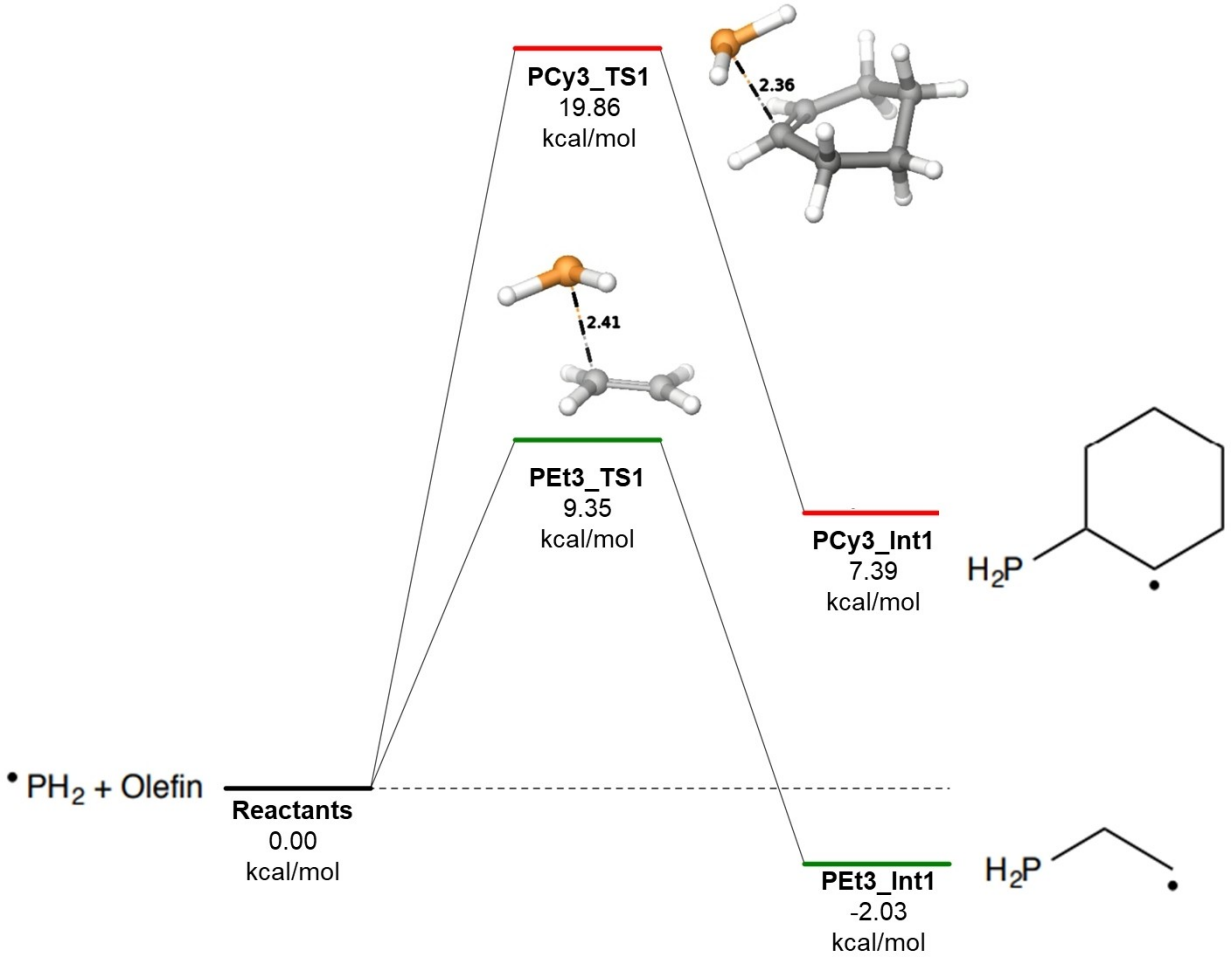
Calculated total free energy for the first alkylation at 298 K and 1 atm.

**Figure 3:**
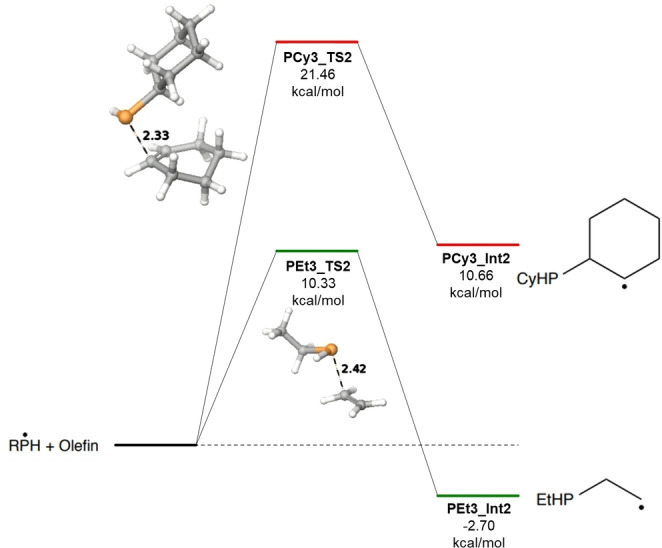
Calculated total free energy for the second alkylation at 298 K and 1 atm.

**Figure 4:**
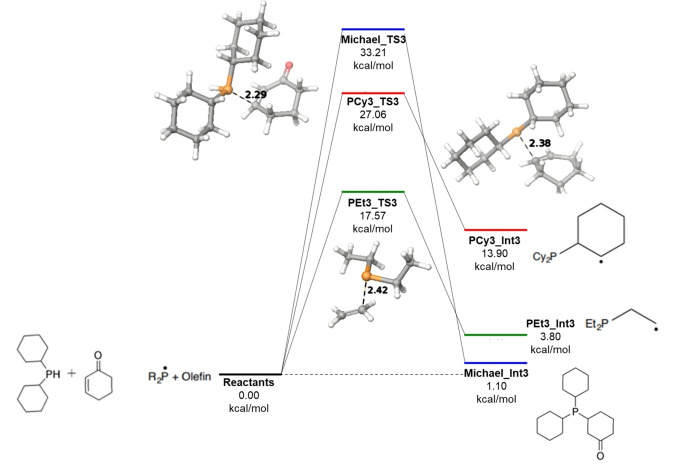
Calculated total free energy for the third alkylation at 298 K and 1 atm.

Results of the calculations highlight the discrepancies in reactivity between the two olefins. On average the three transition states for the radically‐mediated alkylation reactions are ~10 kcal/mol higher and all three additions are overall endothermic for the cyclohexyl derivative (shown in Figures 2, 3, 4). For comparison, the Michael addition step (third alkylation) is plotted along with the two radical alkylation steps in Figure 4 above. Although a higher transition state was located with respect to the reactants, the Michael intermediate is lower in overall free energy for the third alkylation. The elevated energies required and the limitations of temperature in radical‐controlled reactions illustrates the need to switch to a two‐electron mechanism to facilitate the third alkylation for the cyclohexyl system.

## Conclusions

Traditional radical‐catalyzed methods to prepare alkylphosphines are not suitable for the target molecule, tricyclohexylphosphine (**1**). Based on a computational study, elevated energies required for the radical‐catalyzed addition to proceed to the tertiary cyclohexyl derivative cannot be reached experimentally within our system. This necessitated the development of alternative chemistries. Michael addition of dicyclohexylphosphine to 2‐cyclohexen‐1‐one followed by reduction with hydrazine affords tricyclohexylphosphine in good yield but with significant amounts of high‐boiling impurities. The impurities developed in the process are efficiently removed in a recrystallization leading to the desired production in good yield and high purity.

The synthetic route was designed with the manufacturing capabilities available in mind. Although traditional hydrophosphination routes were inaccessible, a safe, low‐cost, high yielding alternative route was achieved, giving reproducible quality at commercial scale.

## Experimental Section

### General considerations and materials

All chemicals and solvents were commercially obtained and used as received unless stated otherwise. Dicyclohexylphosphine (DCHP), **2**, was supplied by Solvay (sold as CYTOP® 266). TGA analysis was run on a Q50 Thermogravimetric Analyzer with Mass Flow Control from TA using a ramp program 10 °C/min up to 500 °C in nitrogen. Effort was made to load samples on the pans and the pans into the furnace under a nitrogen blanket.

### Synthesis of Intermediate (4)

An inerted 1000 mL, 3‐neck round bottom flask was fitted with an addition funnel, reflux condenser, thermowell and magnetic stir bar. Dicyclohexylphosphine (98.8 %, 106.49 g, 0.53 mol, 1 eq.) was added to the reaction flask and set to stir. The DCHP was heated to 60 °C and 2‐cyclohexen‐1‐one (64.36 g, 0.636 mol, 1.2 eq.) was slowly added over ~20 minutes. A very mild exotherm was noted. Following the addition, the reaction mixture was heated to 95 °C and digested for a total of 16 h. During digestion, samples were taken periodically for GC‐FID analysis to monitor the progress of the reaction. Once the reaction was complete, the reaction mixture was stripped to dryness at 140 °C and 4–5 mm Hg over 1.5 h to yield 135.8 g (87 %) of a straw‐coloured solid. The stripped product was dissolved in toluene and analyzed by GC‐FID. Final purity of **4** was 95.3 %.

### Reduction of Intermediate (4)

An inerted 1000 mL, 3‐neck round‐bottom flask was fitted with an addition funnel, a Dean‐Stark apparatus with an overhead condenser and a magnetic stir bar. The flask was charged with **4** (135.4 g, 0.46 mol) and diethylene glycol (555 g) and was slowly heated to 75 °C. Once fully dissolved, hydrazine hydrate (79.88 g, 1.60 mol.) was added via the addition funnel over a period of ~10 minutes. After a 5–10 minute digestion period, KOH pellets (105.35 g, 1.60 mol) were slowly added. The resulting reaction mixture was then digested for ~30 minutes at 75 °C and then heated to 200 °C over a period of 2 h and was digested for an additional 2 h; the water formed (135 g) was removed via Dean Stark apparatus. The mixture was then cooled to 80 °C. Degassed toluene (200 mL) and degassed deionized water (200 mL) were added to the reaction flask, and the resulting reaction mixture was transferred under nitrogen to a 2 L separatory funnel. Phases disengaged within 2 minutes, resulting in a clear yellow organic phase and a cloudy aqueous phase. ^31^P NMR spectroscopic analysis of the aqueous phase showed that **4** was no longer present.

The organic phase (273.2 g) was transferred to a flask equipped with a reboiler, vapor head, takeoff condenser, distillate receiver, nitrogen fill valve, and a vacuum takeoff using a mechanical pump, the reaction mixture was stripped to a maximum of 160 °C and 4 mmHg over a period of 1 h. Upon cooling to ambient temperature, a light yellow solid was obtained (102.2 g, 79 %). ^31^P NMR (toluene‐d_8_): 10.6 ppm (*s*, PCy_3_), 46.1 ppm (*s*, (O)PCy_3_).

### Recrystallization of 1

A saturated toluene solution of crude **1** (48 g) in toluene at 60 °C was charged to a flask. Acetonitrile and methanol (3 : 1 ratio w/w) were added and the mixture was allowed to equilibrate to 60 °C until all solids were dissolved. The mixture was cooled to 5 °C and held for 10 minutes at which point the solids were filtered off, washed with methanol and dried under vacuum. Yield of off‐white solid: 29.8 g (62 %).

## Conflict of interests

The authors declare no conflict of interest.

1

## Supporting information

As a service to our authors and readers, this journal provides supporting information supplied by the authors. Such materials are peer reviewed and may be re‐organized for online delivery, but are not copy‐edited or typeset. Technical support issues arising from supporting information (other than missing files) should be addressed to the authors.

Supporting Information

## Data Availability

The data that support the findings of this study are available in the supplementary material of this article.
