# Supplementary material for: CYTOP® 366: A Tertiary Phosphine Inaccessible by Most Traditional Hydrophosphination Methods
Source: ChemistryOpen. 2024 Jan 3;13(6):e202300264. doi: 10.1002/open.202300264 (PMC11164020; doi:10.1002/open.202300264)
Supplement: Supplementary file 1 — Supporting Information [file OPEN-13-e202300264-s001.pdf]

ChemistryOpen

Supporting Information

CYTOP® 366: A Tertiary Phosphine Inaccessible by Most Traditional Hydrophosphination Methods

Dino Amoroso, Jeff Dyck, Andrew Jackson, Michael Humeniuk, Eleanor Kendrick, Angelo Melaragni, Michael Moser, Izabela Wiater-Protas, Serguei Zavorine, and Jade Markham*

Table of Contents

1. XYZ Coordinates of Optimized Structures 1

1. XYZ Coordinates of Optimized Structures

ethylene

C	0.00000	0.00000	0.66120
C	0.00000	0.00000	-0.66120
H	0.00000	0.92320	1.22690
H	0.00000	-0.92320	1.22690
H	0.00000	-0.92320	-1.22690
H	0.00000	0.92320	-1.22690

Et2P_rad

P	0.25430	-0.01530	0.25960
C	2.04500	-0.09710	-0.17740
C	2.57210	-1.52880	-0.19960
C	0.01690	1.81100	0.14780
C	-1.41140	2.23180	0.48090
H	2.18320	0.38040	-1.15220
H	2.60100	0.50730	0.54580
H	3.63320	-1.55390	-0.44990
H	2.44400	-2.00680	0.77270
H	2.03780	-2.12960	-0.93690
H	0.72920	2.29240	0.82470
H	0.28650	2.12920	-0.86390
H	-1.53250	3.31240	0.39950
H	-2.12500	1.76090	-0.19680
H	-1.67710	1.93860	1.49740

PEt3_Int3

C	4.04760	0.33510	0.85350
C	3.63200	1.73150	0.62080
P	4.64160	2.96210	1.61720
C	6.29890	2.73930	0.81990
C	7.18600	1.72320	1.53420
C	4.08820	4.50180	0.75130
C	4.67300	5.76880	1.36840
H	3.77220	-0.17620	1.76470

H	4.73050	-0.15930	0.17890
H	3.72260	2.00470	-0.43340
H	2.59600	1.89580	0.93130
H	6.15530	2.46840	-0.23040
H	6.78300	3.71890	0.83210
H	8.15240	1.62980	1.03650
H	7.36440	2.02650	2.56660
H	6.72290	0.73600	1.55640
H	4.34570	4.43040	-0.30950
H	2.99750	4.52360	0.81700
H	4.32310	6.65790	0.84210
H	4.38430	5.86020	2.41620
H	5.76330	5.76860	1.32410

PEt3_TS3

P	0.04130	-0.24280	-0.06890
C	1.75100	-0.59450	-0.68550
C	2.78830	-0.68040	0.43410
C	0.28470	1.49630	0.51550
C	-1.04320	2.20080	0.77690
C	-0.66570	0.38750	-2.30010
C	-2.00680	0.55380	-2.46760
H	1.70610	-1.54340	-1.22480
H	2.04060	0.17310	-1.40930
H	3.76780	-0.94960	0.03560
H	2.89110	0.27470	0.95020
H	2.50640	-1.43180	1.17310
H	0.86910	1.45250	1.43910
H	0.88770	2.05300	-0.20850
H	-0.88590	3.19820	1.18890
H	-1.61810	2.30360	-0.14410
H	-1.65150	1.63570	1.48500
H	-0.02510	1.25880	-2.21290
H	-0.18880	-0.51110	-2.67480
H	-2.48760	1.50340	-2.27560
H	-2.64760	-0.27840	-2.72610

CyPH_rad

P	0.62300	2.28400	0.00000
C	-0.92440	-2.14520	0.00000
C	-0.24170	-1.61670	1.25920
C	-0.18990	-0.08900	1.25940

C	0.50590	0.44040	0.00000
C	-0.18990	-0.08900	-1.25940
C	-0.24170	-1.61670	-1.25920
H	-0.77880	2.50180	0.00000
H	-0.92670	-3.23690	0.00000
H	-1.97190	-1.82480	0.00000
H	-0.76140	-1.97090	2.15170
H	0.77950	-2.00910	1.30820
H	-1.21110	0.30810	1.28790
H	0.31820	0.27560	2.15500
H	1.53970	0.07580	0.00000
H	-1.21110	0.30810	-1.28790
H	0.31820	0.27560	-2.15500
H	0.77950	-2.00910	-1.30820
H	-0.76140	-1.97090	-2.15170

Cy2P_rad

P	0.12380	0.16280	0.83490
C	4.33860	-1.49550	-0.58940
C	3.94730	-1.63520	0.88880
C	2.43710	-1.43050	1.09030
C	1.96580	-0.07300	0.52760
C	2.36630	0.06480	-0.95950
C	3.87790	-0.14460	-1.15550
C	-1.13990	4.20380	0.83570
C	-1.79620	4.26730	-0.55110
C	-0.98520	3.46920	-1.58220
C	-0.77760	2.01440	-1.12880
C	-0.10330	1.94580	0.26290
C	-0.92780	2.75110	1.29320
H	5.42230	-1.61070	-0.71110
H	3.86840	-2.30600	-1.16460
H	4.24640	-2.61770	1.27340
H	4.49360	-0.88600	1.47950
H	1.89410	-2.23880	0.57930
H	2.18440	-1.50830	2.15450
H	2.47400	0.72880	1.08730
H	1.81770	-0.68240	-1.55020
H	2.07730	1.04810	-1.34620
H	4.42000	0.66470	-0.64600
H	4.12810	-0.06920	-2.22070
H	-0.16660	4.71350	0.79830
H	-1.74820	4.74200	1.57270

H	-2.81050	3.84760	-0.48900
H	-1.90600	5.30930	-0.87450
H	-1.48290	3.48740	-2.55930
H	-0.00390	3.94580	-1.71910
H	-1.75260	1.50910	-1.07950
H	-0.18200	1.47010	-1.87010
H	0.89100	2.40830	0.18560
H	-0.43310	2.73310	2.27150
H	-1.90670	2.26870	1.42860

PCy3_TS1

Negative frequency = 472.18i

P	0.25990	-0.13240	-0.78110
C	3.91730	-1.20630	-0.08910
C	2.65810	-1.15820	0.71130
C	1.91510	-0.00840	0.89300
C	2.58160	1.35500	0.72440
C	4.08250	1.22310	0.47190
C	4.33730	0.17070	-0.60170
H	-0.07970	1.22770	-0.58680
H	1.24510	0.18190	-1.74550
H	4.71440	-1.63040	0.53370
H	3.79850	-1.90850	-0.92220
H	2.25820	-2.10030	1.06900
H	1.13610	-0.05790	1.64600
H	2.13330	1.91700	-0.09550
H	2.39880	1.93650	1.63050
H	4.49340	2.18960	0.17750
H	4.58860	0.92260	1.39450
H	5.38660	0.15360	-0.89790
H	3.75730	0.42730	-1.49310

PCy3_Int1

P	0.01220	-0.00320	0.02280
C	3.89330	0.69200	-1.47820
C	2.44860	0.83560	-1.13100
C	1.87270	0.02030	-0.02430
C	2.39100	-1.42700	-0.05510
C	3.90830	-1.47750	-0.20800
C	4.34570	-0.76970	-1.48590
H	-0.19880	1.38420	-0.14840
H	-0.18320	-0.29510	-1.34780

H	4.10080	1.16790	-2.43850
H	4.49890	1.23360	-0.73350
H	1.93140	1.73900	-1.43050
H	2.16850	0.45430	0.94490
H	2.07440	-1.94880	0.84990
H	1.93380	-1.94830	-0.90400
H	4.37640	-0.99280	0.65580
H	4.24600	-2.51550	-0.21330
H	3.90080	-1.28010	-2.34540
H	5.42930	-0.82280	-1.60400

PCy3_TS2

Negative frequency = 427.64i

P	-0.03530	-0.07380	-0.26740
C	3.78450	-1.24470	-0.35870
C	2.64350	-1.13870	0.60270
C	1.98140	0.03330	0.89740
C	2.59810	1.36570	0.48110
C	4.07510	1.20580	0.12600
C	4.23370	0.10790	-0.92010
C	0.08270	-1.86640	-2.46150
C	0.62000	-0.53010	-1.94580
C	0.32560	0.59400	-2.94530
C	0.88570	0.26810	-4.32810
C	0.33910	-1.06180	-4.83990
C	0.62950	-2.18820	-3.85180
H	-0.24490	-1.38690	0.21590
H	4.62200	-1.71710	0.16910
H	3.53780	-1.93650	-1.17350
H	2.31420	-2.05680	1.07710
H	1.40480	0.04200	1.81610
H	2.08160	1.78520	-0.38590
H	2.46850	2.08500	1.29130
H	4.47230	2.15120	-0.24700
H	4.64540	0.94340	1.02220
H	5.26670	0.03830	-1.26300
H	3.63120	0.37250	-1.79480
H	0.34180	-2.66320	-1.76030
H	-1.01210	-1.82070	-2.50040
H	1.70730	-0.61910	-1.85640
H	-0.75940	0.73120	-3.01670
H	0.73750	1.53950	-2.58150
H	0.64960	1.07240	-5.02790

H	1.97830	0.20850	-4.26730
H	-0.74440	-0.97400	-4.97540
H	0.76360	-1.29680	-5.81790
H	1.71370	-2.33140	-3.78130
H	0.20790	-3.12930	-4.21140

PCy3_Int2

P	0.19650	0.07000	0.20650
C	3.91220	0.70790	-1.54950
C	2.53180	0.86090	-1.00640
C	2.05080	-0.06060	0.06470
C	2.48350	-1.51040	-0.19620
C	3.96350	-1.60160	-0.56010
C	4.27220	-0.75980	-1.79360
C	-1.17840	-0.07350	2.64230
C	-0.13810	-0.82470	1.80460
C	-0.62400	-2.25240	1.52440
C	-0.93730	-3.00350	2.81640
C	-1.97330	-2.25020	3.64640
C	-1.51080	-0.82390	3.93120
H	0.18690	1.38510	0.72990
H	4.02920	1.29460	-2.46240
H	4.63460	1.12030	-0.82630
H	2.01420	1.80150	-1.15620
H	2.48180	0.24670	1.03120
H	2.27420	-2.11950	0.68590
H	1.88680	-1.91800	-1.02040
H	4.56750	-1.24670	0.28230
H	4.23840	-2.64410	-0.73200
H	3.68840	-1.13730	-2.63860
H	5.32600	-0.84490	-2.06450
H	-0.81630	0.92980	2.87580
H	-2.09260	0.04920	2.04880
H	0.79730	-0.87360	2.37520
H	-1.53150	-2.19500	0.91250
H	0.11290	-2.80090	0.93380
H	-1.29130	-4.01050	2.58660
H	-0.01760	-3.11490	3.40090
H	-2.91820	-2.21750	3.09340
H	-2.17090	-2.77810	4.58150
H	-0.61650	-0.85690	4.56270
H	-2.27410	-0.27990	4.49160

PCy3_TS3

Negative frequency = 478.42i

P	3.89610	0.85310	-1.02800
C	3.31860	-3.49390	0.65920
C	2.47940	-2.38230	1.27900
C	2.46510	-1.13290	0.39810
C	3.88470	-0.63180	0.10240
C	4.72430	-1.75940	-0.50530
C	4.73540	-3.00440	0.37970
C	0.55930	3.05360	0.21870
C	1.13060	4.46470	0.12400
C	2.57490	4.49500	0.61340
C	3.44570	3.48330	-0.12880
C	2.88300	2.06310	-0.03560
C	1.43120	2.05940	-0.54610
C	6.31600	1.38790	0.97100
C	7.39010	2.37340	1.43150
C	7.01870	3.78390	0.98830
C	6.88450	3.88490	-0.53920
C	6.52020	2.59600	-1.20480
C	6.14940	1.44210	-0.53960
H	3.33830	-4.36710	1.31440
H	2.85680	-3.81110	-0.28190
H	1.45670	-2.72510	1.45010
H	2.89570	-2.12310	2.25840
H	1.96000	-1.36070	-0.54770
H	1.88500	-0.35060	0.89010
H	4.33220	-0.33780	1.05910
H	4.30830	-2.01680	-1.48600
H	5.74980	-1.42590	-0.67770
H	5.22670	-2.76460	1.32900
H	5.32580	-3.79190	-0.09310
H	0.50770	2.75340	1.27100
H	-0.46120	3.02800	-0.16950
H	1.09800	4.79250	-0.92060
H	0.51950	5.16540	0.69650
H	2.99540	5.49720	0.50270
H	2.59450	4.25990	1.68340
H	3.52380	3.76850	-1.18550
H	4.45430	3.50410	0.27580
H	2.89050	1.75540	1.01800
H	1.00180	1.05820	-0.49260
H	1.44200	2.33440	-1.60670

H	6.56650	0.37030	1.27570
H	5.37870	1.64150	1.47820
H	7.49280	2.33540	2.51730
H	8.35490	2.08850	1.00180
H	6.07420	4.05470	1.46790
H	7.75800	4.50600	1.33720
H	6.16820	4.67340	-0.80130
H	7.83700	4.21460	-0.96910
H	6.57070	2.57490	-2.28790
H	6.35250	0.51980	-1.07300

PCy3_Int3

P	4.00730	1.24710	-0.85000
C	3.81260	-3.08420	1.07230
C	3.63790	-2.91960	-0.43560
C	4.19550	-1.58000	-0.91700
C	3.55110	-0.41670	-0.15020
C	3.71760	-0.58490	1.36120
C	3.16160	-1.92960	1.82980
C	0.39130	2.78120	0.74620
C	0.36790	4.12090	0.01960
C	1.77170	4.71080	-0.05130
C	2.74280	3.73400	-0.71080
C	2.78820	2.38120	0.01740
C	1.37220	1.79970	0.10370
C	6.72900	0.73270	-0.11970
C	7.97980	1.21750	0.61660
C	8.38970	2.62780	0.20270
C	7.21580	3.60660	0.39860
C	6.01780	3.07900	-0.31500
C	5.55230	1.71480	0.08530
H	3.39480	-4.03790	1.40110
H	4.88240	-3.10750	1.30840
H	4.12470	-3.74030	-0.96670
H	2.57120	-2.97060	-0.67950
H	5.27950	-1.57880	-0.78010
H	4.01590	-1.45640	-1.98730
H	2.48060	-0.48360	-0.37670
H	4.77970	-0.53340	1.62130
H	3.22660	0.23500	1.89260
H	2.08000	-1.94940	1.65560
H	3.31280	-2.04560	2.90510
H	0.68900	2.94550	1.78770

H	-0.60730	2.33930	0.76910
H	-0.01100	3.97310	-0.99750
H	-0.31600	4.81170	0.51710
H	1.76460	5.65380	-0.60200
H	2.12020	4.93690	0.96230
H	2.42920	3.55900	-1.74620
H	3.73920	4.17530	-0.75210
H	3.15250	2.55150	1.03860
H	1.36750	0.87480	0.68170
H	1.02540	1.54750	-0.90610
H	6.93750	0.64310	-1.19120
H	6.46390	-0.26020	0.24290
H	7.78020	1.20560	1.69380
H	8.80130	0.51980	0.44040
H	9.25810	2.95850	0.77650
H	8.67800	2.62760	-0.85280
H	7.00880	3.68090	1.47270
H	7.48450	4.60250	0.04450
H	5.85230	3.39340	-1.34030
H	5.32280	1.72220	1.15980

EtPH_rad

P	-0.34510	-1.02110	0.11080
C	-0.31000	0.81310	-0.10400
C	1.11810	1.34930	-0.12860
H	-1.75490	-1.15090	0.09170
H	-0.83360	1.06330	-1.03030
H	-0.88130	1.26980	0.70820
H	1.12870	2.43180	-0.25890
H	1.63770	1.11630	0.80210
H	1.68770	0.90540	-0.94660

PEt3_TS2

Negative frequency = 387.83i

C	2.38650	-0.13940	0.23540
C	2.66850	0.57600	1.35870
P	4.33260	2.27060	0.88630
C	4.85370	2.51250	2.64930
C	6.30350	2.98140	2.76490
H	1.60530	0.16740	-0.44710
H	2.99320	-0.98640	-0.05590
H	1.96610	1.31870	1.71700

H	3.33540	0.15600	2.10270
H	5.25890	1.23940	0.59690
H	4.18240	3.25840	3.07910
H	4.71080	1.58940	3.21390
H	6.57280	3.15910	3.80750
H	6.98630	2.23210	2.36230
H	6.46230	3.90840	2.21250

PEt3_Int2

C	2.51040	0.16920	0.08750
C	2.86680	1.04840	1.21820
P	4.51830	1.88520	0.90650
C	4.89540	2.52780	2.60250
C	6.33330	3.02830	2.70150
H	2.87400	-0.84760	0.05390
H	2.05790	0.57600	-0.80480
H	2.14320	1.85760	1.33790
H	2.92770	0.49700	2.15770
H	5.30420	0.71110	0.98750
H	4.19650	3.34580	2.79190
H	4.70130	1.75460	3.34770
H	6.53890	3.43480	3.69240
H	7.04210	2.21870	2.51910
H	6.52820	3.81220	1.96830

HPCy2

P	-6.73040	-5.39920	-2.78830
C	-3.42340	-2.11790	-2.71460
C	-3.20670	-3.37040	-1.86890
C	-4.16030	-4.48680	-2.28980
C	-5.62090	-4.02910	-2.21290
C	-5.83340	-2.77300	-3.06100
C	-4.87690	-1.65610	-2.64690
C	-10.86680	-5.51040	-2.35560
C	-11.03450	-4.84240	-0.99410
C	-10.07000	-3.66970	-0.84330
C	-8.62160	-4.10580	-1.06380
C	-8.44180	-4.78120	-2.42210
C	-9.42180	-5.95220	-2.57620
H	-6.59940	-6.23260	-1.64990
H	-2.75590	-1.31820	-2.38740
H	-3.16710	-2.34130	-3.75600

H	-3.38050	-3.12820	-0.81510
H	-2.17270	-3.71170	-1.95110
H	-3.93280	-4.78280	-3.32040
H	-4.01010	-5.37090	-1.66470
H	-5.84960	-3.79190	-1.16990
H	-5.66380	-3.02340	-4.11550
H	-6.86770	-2.42900	-2.98020
H	-5.10960	-1.35060	-1.62120
H	-5.02890	-0.78090	-3.28190
H	-11.53630	-6.36850	-2.44550
H	-11.14820	-4.80150	-3.14150
H	-12.06490	-4.50720	-0.85910
H	-10.83190	-5.57710	-0.20710
H	-10.32520	-2.89860	-1.57830
H	-10.17340	-3.21410	0.14370
H	-7.96140	-3.24090	-0.97480
H	-8.32960	-4.81290	-0.27800
H	-8.66790	-4.05090	-3.20910
H	-9.16820	-6.72320	-1.83880
H	-9.31100	-6.40850	-3.56280

2-cyclohexene-1-one

C	-0.70150	-2.25380	-0.98550
C	-0.37490	-0.92280	-0.31710
C	0.73290	-0.19800	-1.07400
C	1.97370	-1.05310	-1.20170
C	1.75500	-2.50780	-1.34900
C	0.53630	-3.04510	-1.27950
O	3.08840	-0.57600	-1.22330
H	-1.37170	-2.84800	-0.36200
H	-1.23720	-2.08530	-1.92740
H	-1.26510	-0.29680	-0.25790
H	-0.04450	-1.11210	0.70760
H	0.40030	0.02240	-2.09500
H	1.01240	0.74640	-0.60900
H	2.63690	-3.10680	-1.54110
H	0.41700	-4.11110	-1.44430

PEt3_Int1

P	0.01870	-0.13070	0.06450
C	1.86660	0.21850	-0.05190
C	2.36250	0.70730	1.24960

H	-0.10840	-0.89840	-1.11790
H	0.12290	-1.26600	0.89850
H	2.38280	-0.68650	-0.36810
H	1.97750	0.97480	-0.83040
H	2.67370	0.00700	2.01110
H	2.24530	1.74380	1.52990

PEt3_TS1

Negative frequency = 400.97i

P	1.18960	-0.04340	0.30000
C	4.13040	0.80890	-0.34280
C	3.59380	-0.25510	0.31780
H	1.10890	-0.70870	1.54660
H	1.30830	1.22400	0.91330
H	4.31600	1.74680	0.16370
H	4.30920	0.77780	-1.40920
H	3.58370	-0.26070	1.40070
H	3.56170	-1.22510	-0.16160

PEt3_Int3

C	4.04760	0.33510	0.85350
C	3.63200	1.73150	0.62080
P	4.64160	2.96210	1.61720
C	6.29890	2.73930	0.81990
C	7.18600	1.72320	1.53420
C	4.08820	4.50180	0.75130
C	4.67300	5.76880	1.36840
H	3.77220	-0.17620	1.76470
H	4.73050	-0.15930	0.17890
H	3.72260	2.00470	-0.43340
H	2.59600	1.89580	0.93130
H	6.15530	2.46840	-0.23040
H	6.78300	3.71890	0.83210
H	8.15240	1.62980	1.03650
H	7.36440	2.02650	2.56660
H	6.72290	0.73600	1.55640
H	4.34570	4.43040	-0.30950
H	2.99750	4.52360	0.81700
H	4.32310	6.65790	0.84210
H	4.38430	5.86020	2.41620
H	5.76330	5.76860	1.32410

PEt3_TS1

Negative frequency = 386.94i

P	0.04130	-0.24280	-0.06890
C	1.75100	-0.59450	-0.68550
C	2.78830	-0.68040	0.43410
C	0.28470	1.49630	0.51550
C	-1.04320	2.20080	0.77690
C	-0.66570	0.38750	-2.30010
C	-2.00680	0.55380	-2.46760
H	1.70610	-1.54340	-1.22480
H	2.04060	0.17310	-1.40930
H	3.76780	-0.94960	0.03560
H	2.89110	0.27470	0.95020
H	2.50640	-1.43180	1.17310
H	0.86910	1.45250	1.43910
H	0.88770	2.05300	-0.20850
H	-0.88590	3.19820	1.18890
H	-1.61810	2.30360	-0.14410
H	-1.65150	1.63570	1.48500
H	-0.02510	1.25880	-2.21290
H	-0.18880	-0.51110	-2.67480
H	-2.48760	1.50340	-2.27560
H	-2.64760	-0.27840	-2.72610

Et2P_rad

P	0.25430	-0.01530	0.25960
C	2.04500	-0.09710	-0.17740
C	2.57210	-1.52880	-0.19960
C	0.01690	1.81100	0.14780
C	-1.41140	2.23180	0.48090
H	2.18320	0.38040	-1.15220
H	2.60100	0.50730	0.54580
H	3.63320	-1.55390	-0.44990
H	2.44400	-2.00680	0.77270
H	2.03780	-2.12960	-0.93690
H	0.72920	2.29240	0.82470
H	0.28650	2.12920	-0.86390
H	-1.53250	3.31240	0.39950
H	-2.12500	1.76090	-0.19680
H	-1.67710	1.93860	1.49740

Michael_TS3

Negative frequency = 298.47i

P	0.70380	-0.24640	-0.20710
C	5.31060	0.90950	0.01280
C	4.68230	0.21720	1.22920
C	3.34100	-0.45040	0.87560
C	2.34910	0.56370	0.25340
C	2.99290	1.25510	-0.96620
C	4.33310	1.91780	-0.60550
C	0.11450	-0.80600	1.50020
C	-1.36720	-0.47800	1.77110
C	-1.82960	-1.01220	3.13590
C	-1.58220	-2.52010	3.27240
C	-0.10770	-2.85590	3.01080
C	0.34960	-2.33340	1.63770
C	-0.47810	1.86920	-0.68240
C	-0.12930	2.86780	0.41620
C	-1.28700	3.85130	0.66400
C	-2.58570	3.09160	0.96780
C	-2.91210	2.04240	-0.09020
C	-1.82500	1.56220	-0.91930
O	-4.07990	1.64510	-0.24790
H	6.24520	1.40800	0.29750
H	5.57080	0.15110	-0.73990
H	5.36780	-0.53420	1.64050
H	4.51370	0.96080	2.02160
H	3.52160	-1.26690	0.16170
H	2.91610	-0.90250	1.77730
H	2.13770	1.32760	1.01630
H	3.16120	0.50850	-1.75560
H	2.31870	2.01050	-1.38540
H	4.14930	2.73020	0.11280
H	4.77100	2.37860	-1.49950
H	0.71980	-0.28910	2.25890
H	-1.51380	0.60290	1.74110
H	-1.99440	-0.90660	0.97750
H	-1.28130	-0.48420	3.93030
H	-2.89270	-0.78200	3.27840
H	-1.88400	-2.86840	4.26780
H	-2.20820	-3.05610	2.54430
H	0.51430	-2.40130	3.79570
H	0.05570	-3.93950	3.06410
H	-0.21830	-2.84980	0.85080
H	1.40420	-2.58200	1.47560
H	0.17120	1.85330	-1.55530

H	0.08100	2.34660	1.35870
H	0.78700	3.40430	0.15090
H	-1.03580	4.52110	1.49420
H	-1.43050	4.47720	-0.22590
H	-2.50300	2.57440	1.93470
H	-3.44600	3.76360	1.04570
H	-2.10160	0.92340	-1.75380

Michael_Intermediate

P	0.24530	-0.25870	0.00960
C	4.77310	1.15640	-0.34530
C	4.13970	0.32140	-1.45470
C	2.61510	0.40770	-1.39770
C	2.09410	-0.02980	-0.02110
C	2.74120	0.77510	1.10920
C	4.26750	0.71200	1.02380
C	-0.00180	-0.83640	1.76770
C	-1.49100	-0.98180	2.10440
C	-1.70700	-1.52650	3.51600
C	-0.98260	-2.85200	3.71930
C	0.50420	-2.69320	3.42380
C	0.73690	-2.15780	2.01180
C	-0.56320	1.43010	0.11760
C	0.15200	2.54760	-0.65930
C	-0.61360	3.84680	-0.54830
C	-2.06990	3.78690	-0.92500
C	-2.76880	2.62240	-0.21370
C	-2.00140	1.32040	-0.41340
O	-0.08140	4.86770	-0.16790
H	5.86170	1.08450	-0.38870
H	4.51570	2.21020	-0.49930
H	4.49460	0.65290	-2.43270
H	4.44630	-0.72370	-1.33880
H	2.32270	1.43770	-1.60940
H	2.16870	-0.21570	-2.17640
H	2.42790	-1.06940	0.09450
H	2.42150	1.82000	1.05970
H	2.42020	0.39430	2.08020
H	4.58800	-0.31990	1.20470
H	4.70850	1.32520	1.81240
H	0.41210	-0.07480	2.43820
H	-1.99150	-0.01610	2.01800
H	-1.96200	-1.65630	1.37940

H	-1.32940	-0.79760	4.24100
H	-2.77650	-1.63920	3.70570
H	-1.13290	-3.21700	4.73730
H	-1.40660	-3.60280	3.04350
H	0.94090	-1.99510	4.14610
H	1.02490	-3.64510	3.54610
H	0.38890	-2.89970	1.28290
H	1.80890	-2.03190	1.85000
H	-0.59560	1.71760	1.17540
H	1.16460	2.72640	-0.30830
H	0.19240	2.27750	-1.72090
H	-2.53660	4.74650	-0.70880
H	-2.11270	3.61680	-2.00640
H	-2.84120	2.84050	0.85560
H	-3.78710	2.52670	-0.59180
H	-1.95580	1.08080	-1.48120
H	-2.53370	0.49830	0.06450
